# Extraction, quantification and health risk assessment of bisphenol A from various kinds of packaged milk and baby bottles

**DOI:** 10.1016/j.fochx.2025.102387

**Published:** 2025-03-17

**Authors:** Ghulam Mustafa Kamal, Iqra Anwar, Kainat Saadullah, Attila Gere, Samra Yasmin, Jalal Uddin, Abdullah Ijaz Hussain, Gulzar Ahmad Nayik

**Affiliations:** a Institute of Chemistry, Khwaja Fareed University of Engineering & Information Technology, Rahim Yar Khan 64200, Pakistan; b Hungarian University of Agriculture and Life Sciences, Institute of Food Science and Technology Department of Postharvest, Supply Chain, Commerce and Sensory Science, H-1118, Budapest, Villányi út, 29-43, Hungary; c Department of Pharmaceutical Chemistry, College of Pharmacy, King Khalid University, Abha 61441, Saudi Arabia; d Department of Chemistry, Government College University Faisalabad, 38000, Pakistan; e Marwadi University Research Centre, Department of Microbiology, Marwadi University, Rajkot, Gujarat-360003, India

**Keywords:** Bisphenol A, Baby milk, Health risk assessment, Milk packaging, Pasteurization

## Abstract

Bisphenol A (BPA), an endocrine disruptor, is widely used in plastic containers for food packaging. BPA can migrate into food, particularly from polycarbonate (PC) containers, posing health risks by mimicking estrogen. This study investigated BPA concentrations in 23 milk samples from national and local brands in Pakistan, stored in various packaging types: polyethylene terephthalate (PET), polycarbonate (PC), polyethylene (PE), Tetra Pak, and Tetra Brick. BPA extraction was performed using liquid-liquid (LLE) and solid-phase methods, with quantification by HPLC. Raw milk in PC containers showed the highest BPA levels (0.042–0.056 μg/ml at elevated temperatures). BPA was detected in 12 samples, with mean concentrations of 0.019 μg/ml (pasteurized), 0.032 μg/ml (UHT), and 0.049 μg/ml (raw milk). Six baby bottle brands were analyzed, with four showing BPA leaching, and Brand No. 2 exhibiting the highest levels and hazard index. The study emphasizes the need for safer packaging to protect infants from BPA exposure.

## Highlights

• BPA migration analyzed in 23 milk samples from various packaging types.
• Highest BPA leaching observed in polycarbonate containers for raw milk.
• HPLC detected BPA in 12 milk samples with varying concentrations.
• Baby bottles showed BPA leaching, with polycarbonate posing higher risks.
• Safer, BPA-free packaging recommended for infant nutrition and health.

## Graphical abstract

**Figure:**
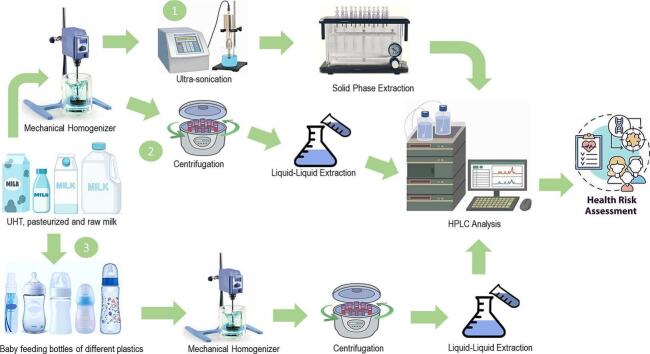

## 1 Introduction

Packaging is necessary for food protection, keeping both its safety and quality by providing a barrier against components that cause microbiological decomposition (Vilarinho, Sendón, Van der Kellen, Vaz, & Silva, 2019). The importance of food packaging lies in its ability to protect food from external factors that can compromise its quality and safety, such as temperature, physical damage, microbes, and odors, thereby extending its shelf life and preventing food loss. Due to the migration of numerous chemicals from packaging into the food, such as polymers, monomers, and processing aids, plastics could be a source of chemical contamination in food (Manoli & Voutsa, 2019). Due to the serious health concerns of these leaching chemicals, it is necessary to detect and ensure their concentrations into the food materials.

The development of innovative packaging technologies that ensure the quality and safety of food has been driven by changes in consumer demand, commercial production trends, selling procedures, and consumer lifestyles. These changes aim to provide fresh, nutritious, and high-quality food products that are easily accessible and have an extended shelf life. The 20th century witnessed several advancements in packaging technology, including the development of intelligent or innovative packaging (Han, Ruiz-Garcia, Qian, & Yang, 2018). Packaging not only improves food safety but also facilitates the transportation of packaged foods over long distances (Marsh & Bugusu, 2007). The quality and raw materials of the packaging materials are very important. The low-grade plastics and specially polycarbonate based plastic packing materials are more prone to be the source of BPA leaching into the foods.

Pakistan has also been facing a number of health issues, most of which are related to poor diet and hazardous environments, since pollutants and poisonous substances enter the food chain and affect the health of organisms. In emerging countries, increased milk and dairy products consumption yield significant nutritional benefits and its packaging has become a crucial issue for the health of the consumers. Growing societal concern over food contamination with endocrine disruptors has emerged recently. These substances, known as EDs, can infiltrate food through various pathways, notably via plastic packaging. Incomplete processing in chemical synthesis and plastic production can lead to undesired chemical residues (Liao & Kannan, 2013; Taskeen, 2012).

Endocrine disruptors, as defined by the U.S. Environmental Protection Agency (USEPA), are defined as “an exogenous agent that disrupts the production, release, transport, metabolic processes, binding action, or elimination of natural hormones in the body responsible for the maintenance of homeostasis and the regulation of developmental processes”. Plastic packaging comes in different grades: high-grade plastics, often BPA-free, help mitigate the risk of bisphenol leaching into stored food or liquids. In contrast, low-quality plastics are more prone to containing bisphenol and other harmful substances (de Almeida Soares et al., 2023; Kavlock et al., 1996).

BPA, widely utilized in plastic production, is the most extensively studied among various bisphenols. BPA is a small, symmetrical molecule with two phenolic rings connected by a methyl bridge and two methyl groups attached. Bisphenol A, a xenoestrogen, mimics estrogen by binding to both α and β estrogen receptors due to its structure with two -OH groups on benzene rings. Some studies reported the toxicity order of these bisphenols is in the following order: BPAF > BPA = BPF > BPS > BPC > BPB > BPAP > BPZ. BPA is a comparatively tiny, symmetric molecule having two phenolic rings connected by a methyl bridge and two functional methyl groups attached (Catron et al., 2019; Pelch et al., 2019).

Bisphenol A (BPA) is a contentious chemical used primarily in the manufacture of polycarbonate plastics (PC) and epoxy resins, which are commonly found in food and drink packaging today. BPA leaches into liquid food from polycarbonate containers via hydrolysis of the ester bond with aqueous food and diffusion into dry food. Ethanol-water solutions can enhance BPA solubility by depolymerizing polycarbonate, contributing to BPA presence in various plastic containers. Due to this, BPA closely resembles 17 beta-estradiol structurally, it can bind to estrogen receptors (ER) and disrupt hormonal function. However, the plastics used in many baby bottles and milk containers contain Bisphenol A, a chemical present in food packaging, has the ability to potentially migrate into the contents and create health hazards for consumers. Plastic containers commonly used for storing milk or other foods, including baby bottles, Tetra Pak packaging, and local plastic bags, may carry a risk of bisphenol exposure. Compared to adults, babies are more susceptible to exposure to BPA. These days, plastic infant bottles are generally accessible in a range of sizes, brands, and costs. Eighty percent of newborns' BPA exposure comes from the leaching of BPA from these plastic bottles, which is the main source of exposure (Almeida, Raposo, Almeida-González, & Carrascosa, 2018). Just under 1 % of exposure usually results from powdered formula, with the remaining 19 % coming from liquid formula kept in polycarbonate (PC) cans or containers. (Almeida et al., 2018; Yun, Ho, Tan, & How, 2018).

Production of polycarbonate (PC) and epoxy-phenolic resins involves the use of chemical monomers such as bisphenol A (BPA). Packaging and the food sector frequently utilize BPA as a plasticizer because PC, which has strength, durability, and low density, is a typical material for food containers and infant bottles. Daily milk consumption raises concerns about the development of young children, as polycarbonate infant bottles are prone to BPA exposure, especially when sterilized or in contact with hot liquids. BPA can migrate from baby bottles into the milk, particularly when the bottles are heated or exposed to acidic or fatty substances. Additionally, surface scratches or wear from repeated use can heighten the risk of BPA leaching (Le, Carlson, Chua, & Belcher, 2008; Yun et al., 2018).

Studies have investigated factors affecting BPA release, including heating, plastic types, food pH, fat content, container aging, temperature, resin manufacturing, UV radiation, salt presence, and canned food storage duration (Baz et al., 2023; Kumar, Gupta, Tomer, Kaur, & Kumar, 2018).Long-term consumption of contaminated food can result in fetal development issues, preeclampsia, miscarriages, endocrine disruption, obesity, neurological disorders, thyroid problems, cancer, and reproductive issues (Godswill & Godspel, 2019; Pelch et al., 2019).

Liquid-Liquid Extraction is the conventional method that involves partitioning between two immiscible liquid phases and popular for its simplicity, low cost, and ability to extract BPA efficiently from aqueous solutions. In contrast to this Solid Phase Extraction is a more advanced and environmentally sustainable technique, involving the retention of BPA on a solid adsorbent and offers higher precision, better reproducibility, and reduced solvent use compared to LLE. SPE is particularly advantageous for analyzing BPA in complex samples such as milk, water, and biological fluids due to its ability to concentrate BPA and minimize matrix interferences (Yıldırım, Gürkan, & Altunay, 2017).

The study aimed at extracting, analyzing and assessing health risk of the BPA leached from various plastic packaging materials commonly used in the milk industry and baby bottles. Main focus is on the extent of migration of BPA into the milk stored in various packages available in local Pakistani market and explore the effect of factors such as temperature, storage duration, and packaging conditions that influence the release of BPA into the packed milk samples. Both the LLE and SPE extractions techniques were employed for the extraction of BPA from milk samples and HPLC is used for the quantification. We also investigated the vulnerability of the BPA leaching in different types of milk; i. e; UHT, raw milk, pasteurized etc. stored in various plastic packaging. With an emphasis on BPA leaching from plastic containers in specific storage duration and analysis temperature, this study also attempts to assess the presence and amounts of BPA in a range of infant bottles and the health risk assessment associated to these baby bottles is the most are crucial element of analysis. Various brands of plastic baby bottles were considered and dietary patterns were assessed to estimate the levels of bisphenol leaching.

## 2 Materials and methods

### 2.1 Sample collection of milk stored in various plastic packaging

A careful selection of 23 samples was done in total, from which 19 milk samples were collected from popular brands, available in Pakistani supermarkets and 4 fresh-raw milk samples were collected randomly from local dairy farmers. The milk samples of various dairy brands were packed in plastic bottles, plastic wrappers, Tetra Pak, or Tetra Brick cartons while the fresh-raw milk samples were taken in glass bottles. Sample's packaging materials included the polyethylene (PE), Tetra Pak and Tetra Brick boxes (had an interior layer of PE), PHDE and PET while the raw milk samples were stored in polycarbonate (PC) storage tanks for ten days. All the collected samples were provided the same storage duration i.e.; ten days, by keeping in view the manufacturing date of all the branded milk samples. The collection was focused on the composition of packaging's to ensure that how different materials contribute to BPA leaching into the stored product. Another point of consideration during the sample selection and collection process was the different sterilization and processing methods (e.g., UHT, pasteurization) adopted by 19 dairy brands, thus eight UHT milk samples and eleven pasteurized milk samples were taken to identify how processing impacts BPA migration from the packaging into the stored milk. Until analysis, all milk samples were kept at 4 ± 1 °C; in their original packaging and transported to lab, to ensure consistency and reproducibility of the study. Nineteen branded milk samples and two raw milk samples were melted at room temperature before analysis, while the remaining two raw milk samples were subjected to heat treatment in the warm water bath at 70 °C for one hour. Moreover, all the apparatus used was made of standard glass, complying the no contact of the samples with any exterior plastic source. The pasteurized milk samples' expiry date was 1 to 8 days later, whereas the UHT milk samples' expiry date was at least 1 month later at the time of analysis.

### 2.2 Collection and Preparation of baby bottles samples

Famous brands of six plastic baby bottles widely sold in Pakistani market were also collected for examining the extent of BPA migration. Brand new and gently used both kinds of baby feeding bottles were taken and labelled carefully (as 1,2,3,4,5,6) to get the accurate analysis. BPA free baby feeding bottles were also used in sample collection. Baby feeding bottle # 1and 2 were gently used and bottle # 3,4,5 and 6 were brand new. Among these collected baby bottles, bottle # 5 and 6 were claimed as BPA free by manufacturer. Overall, six milk samples were collected carefully to store into the collected baby feeding bottles. The milk samples were collected randomly from the local supermarket and from the local dairy farmers. Four milk samples were of famous international milk brands, and who claimed that their products are packed in BPA free packaging, were collected and immediately transferred to the glass bottles and kept in ice-buckets until transported to lab. Among these four milk samples, three were of pasteurized milk and one was UHT milk. All four milk samples were having same manufacturing date of two days earlier and expiry date of almost one month later. The remaining two samples utilized for the analysis of BPA leaching from the baby bottles, were of fresh raw milk, that were taken directly from different farmers in the glass bottles and kept in ice-buckets and brought to the lab freezer. All the milk samples were taken to the lab afterwards to ensure that these were not in contact with any plastic material and kept frozen at 4 ± 1 °C until further processing. All the baby bottles were sterilized before analysis and the milk samples (in glass bottles) were melted at 25 °C in warm water bath and filled in the baby bottles and kept them stored for 2 months at 4 ± 1 °C in the freezer in order to give enough time for BPA leaching from these baby bottles into the milk. Baby bottle # 1, 2 (gently used bottles) and # 3 (brand new) were filled with pasteurized milk while Baby bottle # 4 (brand new) and # 5 (brand new, BPA free) were filled with the raw milk samples and bottle # 6 contained the UHT milk sample. The raw materials of the baby bottles used to store the milk samples were polyethylene terephthalate (PET) for Bottle #1 and #3, while for bottle #2 was polycarbonate (PC) and bottle #4 was polyvinyl chloride (PVC) and bottle #5 and #6 was high-density polyethylene (HDPE).

### 2.3 BPA extraction from various packaged milk samples using LLE

Each milk sample was homogenized using a mechanical homogenizer. A 5 ml aliquot of each homogenized milk sample was taken and put into a 50 ml PYREX™ round bottom; ungraduated centrifuge tube made of Borosilicate glass. 5 ml of acetonitrile was added to each tube, and vortexed for 5 min. The mixture was centrifuged at 3000 rpm for 10 min. A new centrifuge tube was filled with the upper layer (acetonitrile). Using n-hexane, the acetonitrile extract was further purified. Following a 5-min vortex, 5 ml of n-hexane was added to each tube. For 10 min, the mixture was centrifuged at 3000 rpm. In a new centrifuge tube, the top layer (n-hexane) was added. Under a mild stream of nitrogen gas, the n-hexane extract was evaporated until it was dry. The dried n-hexane extract was reconstituted in 5 ml of acetonitrile. Using HPLC, the level of BPA was determined (Rostamzadeh, Nemati, Farajzadeh, & Ashar Mogaddam, 2021).

### 2.4 BPA extraction from various packaged milk samples using SPE

Using a mechanical homogenizer, each milk sample was homogenized. A 10 ml aliquot of each homogenized milk sample was collected and mixed with 5 ml of methanol. The addition of methanol aimed to destabilize the milk's emulsion. A two-step approach was used to assure homogeneity and enable analyte extraction: (1) device vortex agitation for 1 min using a Vortex ZX4 shaker, and (2) sonication in an ultrasonic bath for 15 min at room temperature using a Branson 2210R-MT Ultrasonic. The sample was diluted with deionized water to 100.0 ml after sonication. The addition of water aimed to reduce the viscosity of the sample, facilitating better flow during solid phase extraction (SPE). After the sample preparation done, the solution was passed through a 0.45 μm membrane filter. The solid-phase C-18 cartridge (Merck, LiChrolut® RP-18 (40–63 μm) 500 mg 3 ml standard PP-tubes) was conditioned sequentially with 5 ml of methanol and 10 ml of deionized water, preventing the cartridges from becoming dry. The filtrate was run through the cartridge, rinsed with 20 ml of water to remove any sugars and other polar milk components, washed with water/methanol (80:20, *v*/v) to eliminate interferences and then finally eluted with 5 ml of HPLC-grade methanol. The eluate was dried by vacuum followed by reconstituted in 1 ml of methanol, filtered through a 0.45-μm syringe filter and then injected into the HPLC system. Under such conditions, the BPA peak was well-separated from interferences and had a retention time of around 5 to 6 min (Hadjmohammadi & Saeidi, 2010).

### 2.5 Milk Sample Preparation for Analysis of BPA Leaching from Baby Bottles

To prepare the milk samples for assessment of baby feeding bottles, each baby bottle containing the stored milk was treated at different working temperatures for one hour. Sample 1 and 2 were melted at 36 °C, sample 3 and 4 were heated at 40 °C and sample 5 and 6 were heated at 45 °C in a warm water bath for one hour and each milk sample was homogenized using a mechanical homogenizer. A 5 ml aliquot of homogenized milk sample was taken from each baby feeding bottle and placed in a 50 ml centrifuge tube. Then 5 mL of acetonitrile (ACN) was added to each tube, and the subsequently, the mixture was centrifuged for 10 min at 3000 rpm to separate the milk sample from the solvent. The upper layer, containing acetonitrile, was carefully transferred to a new centrifuge tube.

### 2.6 Liquid-Liquid Extraction and Purification of Samples Taken from Baby Bottles

For further purification, liquid-liquid extraction was done using a solvent n-hexane. To each tube containing the acetonitrile extract, 5 ml of n-hexane was added, and the mixture was vortex for 5 min. After vortex, the tube was centrifuged for 10 min at 3000 rpm to separate the layers. A fresh centrifuge tube was used to transfer the top layer, which contained n-hexane. The n-hexane extract was evaporated to dryness using a moderate stream of nitrogen gas to concentrate it. To analyze the presence of BPA, the dried n-hexane extract was reconstituted in 1 ml of acetonitrile. BPA levels were determined using a HPLC. The quantification of BPA in each milk sample was achieved by comparing the results with standard calibration curves.

### 2.7 HPLC Instrumentation and Chromatographic Conditions

Liquid chromatographic analysis of bisphenol-A was conducted at TTI Labs Lahore Pakistan using a quaternary high-pressure pump (1110 series) and an autosampler. With an isocratic program, chromatographic separations were achieved on a C18 column (2.6 μm, 4.6 × 100 mm). The analysis was performed at room temperature (20 ± 2 °C). HPLC determination of BPA was carried out at a flow rate of 0.5 ml/min with an injection volume of 10 μL, utilizing a mobile phase consisting of water and methanol (30:70, *v*/v). A UV detector was applied to detect the analyte at a wavelength of 270 nm. The quantification of BPA in each milk sample was achieved by comparing the results with standard calibration curves.

### 2.8 Health Risk Assessment of Bisphenol in Baby Feeding Bottles

Human health risk assessment includes hazard identification, toxicity evaluation, exposure evaluation, and risk characterization; it examines the possibility of adverse health effects associated with contaminant exposure over a specific population and a specified period of life (Luo, Yin, Dang, & Liu, 2018).

### 2.9 Hazard identification

Health risk associated with BPA ingestion by using plastic baby bottles in the 2 age groups 0 to 6 and 6 to 12 months was estimated according to the data collected from several cities of Pakistan (Dehdashti, Nikaeen, Amin, & Mohammadi, 2023).

### 2.10 Chronic Daily Intake

The Chronic Daily Intake (CDI) formula used to calculate health risk assessment in milk is typically expressed as.

(1) $$\mathrm{CDI}=\frac{\text{Conc}.\times \mathrm{IR}\;}{\mathrm{BW}}$$

• C represents the concentration of the contaminant in the milk,

• IR is the ingestion rate (amount of milk consumed per day), and

• BW stands for body weight.

This formula considers the concentration of the contaminant in the milk, the daily intake rate of milk, and the body weight of the individual. By multiplying the concentration by the ingestion rate and dividing by body weight, the CDI provides a measure of exposure to a contaminant through milk consumption, aiding in assessing health risks associated with that exposure (Zyambo et al., 2022). Chronic daily intake reference values are presented in Table 1.

**Table 1:** Chronic daily intake reference values.

**Age groups**	**Daily Intake**	**Body weight**	**References**
**0–6 months**	191.1 μg/kg/day		(Dehdashti et al., 2023).
Female		7.3 kg	(Fenton, 2003)
Male		7.9 kg	
**6–12 months**	161.37 μg/kg/day		(Dehdashti et al., 2023).
Female		8.9 kg	(Fenton, 2003)
Male		9.6 kg	

### 2.11 Dose response and risk characterization

HI was used to evaluate the non-carcinogenic effects of BPA due to its oral exposure. Where HI˂1 indicates safety levels and HI˃1 indicates potential risk. The HI value was calculated using eq. (2).

(2) $$\mathrm{HI}=\frac{\mathrm{CDI}}{\mathrm{RFD}}$$

Chronic Daily Intake (CDI) is a measure of exposure to a contaminant (BPA) through milk intake. The CDI values calculated earlier in the study were utilized in this formula to calculate HI. BPA reference dose (RFD) based on the oral exposure route was considered as 50 μg/kg/day using the USEPA Integrated Risk Information System (IRIS) database (Dehdashti et al., 2023).

### 2.12 Estrogenic Activity Assessment

It has been shown that BPA has less estrogenic action than natural oestrogens. However, the elevated levels in infant bottles might result in a significant increase in estrogenic potency.

Due to the estrogenic activity of BPA, the equation was used to determine the estrogen equivalency (EEQ).

(3) EEQ=EP×C

where C is the amount of BPA (μg/L) in the baby bottles and EP is the estrogenic potency of BPA. Particularly, μgE2/L is the EEQ unit. An element possesses less estrogenic activity when its EP is less than 1 (Dehdashti et al., 2023).

## 3 Results and discussion

### 3.1 Quantification of BPA in Milk from Various Packaging Materials Using HPLC

A total of 23 samples were collected and analyzed, with BPA being detected in 12 milk samples. The milk samples, which included raw milk, pasteurized milk, and UHT milk, were stored in various types of plastic containers such as polycarbonate (PC), polyethylene (PE), and Tetra Pak. The analysis focused on the leaching of BPA from these packaging materials into milk when provided the same storage duration and analysis temperature. LLE was performed to extract BPA from twelve samples while for remaining eleven samples SPE was employed. The primary objective of employing both extraction techniques was to conduct a comparative analysis to evaluate their effectiveness in extracting our target analytes. LLE has been commended for its versatility making it suitable for a wide range of sample matrices but it is a little time consuming and having environmental concerns. On the other hand, SPE offers advantages such as greater recoveries, less time consumption and less solvent usage with more consistent results (Kim, Do, Yeh, & Cunningham, 2014).

By utilizing both extraction techniques LLE and SPE, their performance can be assessed and a comparative approach can be established to identify the most effective method for BPA extraction and to enhance the reproducibility of our results.

Quantification of all the milk samples whether prepared by LLE or SPE was done using HPLC and are presented in Table 2.

The results exhibited that the brand 9 and brand 3, showed the highest BPA levels i.e.; 0.056 (μg/ml) and 0.042 (μg/ml). Both of these samples were stored in PC containers carrying raw milk and were subjected to elevated temperature conditions. This finding clearly indicates an evident-direct relationship between increased BPA migration from PC containers and high temperature conditions. Based on these analyzed results, it is apparent that BPA leaching levels varies among the different types of milk packaging. Among those 10 samples that were analyzed at 25(°C) and BPA extraction was done using LLE results revealed that the UHT milk, pasteurized milk and raw milk, were found to be contaminated with BPA, brand1, brand 4, brand 7 and brand 10 with concentrations 0.038 μg/ml, 0.019 μg/ml, 0.026 μg/ml, 0.032 μg/ml respectively. Only brand 4 containing the pasteurized milk exhibited a low concentration of BPA among the LLE prepared samples. While all the other pasteurized milk samples and the raw milk samples showed no BPA leaching at room temperature regardless of their packaging materials.

HPLC analysis conducted on the remaining 11 milk samples in which extraction of BPA was done using SPE are also presented in Table 2. This analysis revealed that both pasteurized and UHT samples exhibited BPA contamination at notably low concentrations and it becomes apparent that the BPA levels were lower in pasteurized milk as compared to UHT milk, consistent with findings from the extraction of using LLE method. The results revealed that both the pasteurized milk, as well as UHT milk among the analyzed samples, majority were found BPA contaminated, with concentrations 0.0017 μg/ml, 0.0022 μg/ml, 0.0043 μg/ml, 0.0063 μg/ml, 0.0051 μg/ml and 0.0073 μg/ml shown by brand 11, brand 13, brand 14, brand 15, brand 18 and brand 21 respectively but with a low limit of detection. Additionally, some milk samples were observed to have no BPA contamination.

**Table 2:** BPA concentrations in various milk brands extracted using LLE and SPE.

Sr.no	Sample	Plastic Type	Milk Type	Storage Temp (°C)	Working Temp (°C)	Conc. of BPA (μg/ml)
Elaborating BPA levels in various milk brands extracted using LLE						
1	Brand 1	Tetra pack	UHT Milk	4	25	0.038 ± 0.001
2	Brand 2	PE	Pasteurized	4	25	ND
3	Brand 3	PC	Raw Milk	4	70	0.042 ± 0.0025
4	Brand 4	PE	Pasteurized	4	25	0.019 ± 0.001
5	Brand 5	Tetra pack	UHT Milk	4	25	ND
6	Brand 6	PE	Pasteurized	4	25	ND
7	Brand 7	Tetra pack	UHT Milk	4	25	0.026 ± 0.006
8	Brand 8	Tetra pack	UHT Milk	4	25	ND
9	Brand 9	PC	Raw Milk	4	70	0.056 ± 0.001
10	Brand 10	Tetra pack	UHT Milk	4	25	0.032 ± 0.001
11	Brand 11	PC	Raw Milk	4	25	ND
12	Brand 12	PC	Raw Milk	4	25	ND

Elaborating BPA levels in various milk brands extracted using SPE						
1	Brand 11	PE	Pasteurized	4	25	0.0017 ± 0.0001 < LOD
2	Brand 12	PE	Pasteurized	4	25	ND
3	Brand 13	PE	Pasteurized	4	25	0.0022 ± 0.002 <LOD
4	Brand 14	PE	Pasteurized	4	25	0.0043 ± 0.001 < LOD
5	Brand 15	Tetra pack	UHT Milk	4	25	0.0063 ± 0.00095 < LOD
6	Brand 16	Tetra Brick	Pasteurized	4	25	ND
7	Brand 17	PE	Pasteurized	4	25	ND
8	Brand 18	PE	Pasteurized	4	25	0.0051 ± 0.0002 <LOD
9	Brand 19	Tetra pack	UHT Milk	4	25	ND
10	Brand 20	HDPE	Pasteurized	4	25	ND
11	Brand 21	Tetra pack	UHT Milk	4	25	0.0073 ± 0.0004 < LOD

*LOD: Limit of Detection, *ND: Not Detected.

Table 3 provides details on the contamination ranges, mean, and median values of BPA across the 23 tested samples, determined using HPLC with a UV–visible detector. In this study, the ranges of BPA in raw milk, UHT, and pasteurized milk samples were revealed 0.00–0.056 μg/ml, 0.0063–0.038 μg/ml, and 0.0017–0.019 μg/ml respectively. The mean concentration of BPA in raw milk samples treated at elevated temperature was (0.025 μg/ml) that was higher than that in UHT milk i.e.: (0.022 μg/ml), followed by pasteurized milk i.e.:(0.007 μg/ml) and the least BPA i.e.: (0.00 μg/ml) was found in raw milk samples that were analyzed at similar temperature conditions with UHT and pasteurized milk samples.

**Table 3:** Overall migration statistics of BPA from containers into the various kinds of milk samples.

**Sr. No**	**Samples**	**No. of samples**	**Mean**	**Median**	**±SD**	**RSD**
1	Raw milk from storage tanks (high temp)	02	0.049	0.049	0.0099	20.20
2	Raw milk from storage tanks (room temp)	02	0.00	0.00	0.00	0.00
2	Pasteurized milk (room temp)	11	0.007	0.0043	0.0071	110.7
3	UHT milk (room temp)	08	0.022	0.026	0.014	65.90

The frequency of BPA detection among the different types of analyzed milk samples is expressed in Table 3. The statistics showed the BPA identified in milk samples (pasteurized, UHT, raw milk) stored in various plastic containers (PE, Tetra Pak, PC). Moreover, it is highlighted that raw milk in PC packaging, demonstrated significantly higher BPA migration (under elevated temperature conditions) as compared to the raw milk samples analyzed at room temperature and also with all the other UHT and pasteurized milk samples. Additionally, it is evident that UHT milk packaging consistently displayed the higher BPA levels compared to pasteurized milk, as observed in both the LLE and SPE methods.

### 3.2 Comparison of Vulnerability of BPA Leaching Among Various Milk Samples

Current analysis revealed that the extent to which the various milk samples are vulnerable to the migration of BPA from the packaging material. The highest BPA levels were found in raw milk, followed by UHT Milk, and then Pasteurized Milk. The vulnerability of various milk types towards BPA migration is presented in Table 4. Thus, it is confirmed that the raw milk (PC containers) is the most vulnerable to BPA contamination, under any kind of storage conditions.

**Table 4:** Frequency of vulnerability of various types of milk Samples and Positive Sample via both extraction techniques LLE and SPE.

**Sr.no.**	**Samples**	**Total No. of samples**	**Positive samples**	**Mean concentration** **(μg/ml)**
**Frequency of vulnerability of various types of milk Samples (LLE)**				
01	Pasteurized milk	3	1	0.019
02	UHT milk	5	3	0.032
03	Raw milk under specific storage conditions	4	2	0.049

**Frequency of vulnerability of various types of milk Samples (SPE)**				
01	Pasteurized milk	8	4	0.0033
02	UHT milk	3	2	0.0068

It is found that the frequency of vulnerability of BPA leaching among samples analyzed by SPE; the pasteurized milk and UHT milk showed the mean concentrations 0.0033 μg/ml,0.068 μg/ml respectively from low to high. While the vulnerability of BPA leaching among samples analyzed by LLE, pasteurized milk, UHT milk and raw milk demonstrated the mean concentrations of BPA as 0.019 μg/ml, 0.032 μg/ml and 0.049 μg/ml respectively. Additionally, it is evident that UHT milk packaging consistently displayed the highest BPA levels i.e.; (mean concentration: 0.032 μg/ml) as compared to pasteurized milk i.e.: (mean concentration: 0.019 μg/ml), as observed in both the LLE and SPE methods.

The most significant finding of the current study was that the raw milk packaging (PC) demonstrated the highest BPA migration (under specified conditions) i.e.; 0.049 μg/ml as compared to all the other types of milk and plastic containers as well. Quantitative analysis was conducted using an HPLC-UV detector. Our findings agree with the BPA from materials intended for food contact (packaging and storage containers) is influenced by factors such as the type of material, pH, lipid content of the food, temperature, and duration of contact. Moreover, our study observed significant variations in BPA levels when raw milk was exposed to elevated temperature. The BPA leaching into raw milk from PC containers increased manifolds when provided temperature was raised from 25 °C to 70 °C.

### 3.3 Estimation of BPA Leaching into milk from various brands of baby bottles

Investigation of Bisphenol A (BPA) and its derivatives concentration leached from baby feeding bottles in Pakistani consumers were focused. High-Performance Liquid Chromatography (HPLC) to quantify BPA concentrations in various milk samples that were kept in baby bottles for 2 months at different temperatures. Results of the HPLC analysis of milk stored in various brands of baby feeding bottles are detailed in Table 5.

**Table 5:** Quantification of BPA in milk samples kept in various brands of baby bottles subjected to different analysis temperatures.

**Brands**	**Type of milk**	**Retention time**	**Storage Temperature**	**Analysis Temperature**	**Concentration (μg/ml)**
1	Pasteurized milk	5	4 °C	36 °C	0.16
2	Pasteurized milk	5	4 °C	36 °C	2
3	Pasteurized milk	5	4 °C	40 °C	0.040
4	Raw milk	5	4 °C	40 °C	0.018
5	Raw milk	5	4 °C	45 °C	ND
6	UHT milk	5	4 °C	45 °C	ND

ND = Not detectable.

The table presents the concentration of Bisphenol A (BPA) measured in micrograms per milliliter (μg/ml) for six different brands. Results obtained for the analyzed brand samples exhibited negligible amount of BPA in most of the samples as the response was below the limit of detection or ND for these samples, indicating they had no BPA content so these Brands are safe for babies (Liao & Kannan, 2013) except from sample 2, in which there is maximum concentration of BPA was found that is 2 μg/ml.

### 3.4 Health risk assessment

#### 3.4.1 Chronic daily intake

The chronic daily intake of bisphenol A in the literature is reported to be less than 1 μg/kg BW/day. To compare the chronic daily intake of Bisphenol A (BPA) with the concentrations provided in the Table 6 for different age groups (0–6 months and 6–12 months).

**Table 6:** Chronic Daily Intake of milk stored in various brands of baby bottles.

**Brand Names**	**Age Groups**	**EDI (μg/kg/day)**
	**0–6 months babies**	
Brand 01	Female	4.18
	Male	3.87
Brand 02	Female	52.3
	Male	48.37
Brand 03	Female	1.04
	Male	0.96
Brand 04	Female	0.47
	Male	0.43
Brand 05	Female	0
	Male	0
Brand 06	Female	0
	Male	0
	**6–12 months babies**	
Brand 01	Female	3.43
	Male	3.18
Brand 02	Female	42.94
	Male	39.81
Brand 03	Female	0.85
	Male	0.79
Brand 04	Female	0.38
	Male	0.35
Brand 05	Female	0
	Male	0
Brand 06	Female	0
	Male	0

These findings highlight variations in Bisphenol content among the analyzed milk samples, likely influenced by factors such as milk sources, baby feeding bottles of different brands, and storage conditions. Various studies the raw milk from the baby bottles had the highest mean content of BPA (0.265 μg/L) among the analyzed samples. The raw milk sample from the storage baby bottles showed the highest mean BPA concentration (0.265 μg/L) of all the samples examined. It is clear from comparing this to the chronic daily intake data for several brands that none of them had chronic daily intake levels that are higher than this mean BPA concentration. Although the average BPA amount of raw milk is not exceeded by any of the brands, it's crucial to remember that newborns, particularly those between the ages of 0 and 6 months, may have lower tolerance levels for BPA because of their developing systems. Brands that have higher levels of chronic daily use may be more responsible for infants' total BPA exposure. Consequently, it's critical that parents and the responsible authorities should understand possible BPA exposure sources and take mitigation or alternative measures into consideration.

#### 3.4.2 Hazard Index

Hazard index of milk stored in baby bottles of various brands was estimated in babies of 0–6 months of age and 7–12 months of age. Results are presented in Table 7. RFD reference dose based on the oral exposure route was considered as 50 μg/kg/day using the USEPA Integrated Risk Information System database (Dehdashti et al., 2023).

**Table 7:** Hazard Index of milk stored in various brands of baby bottles.

**Brand names**	**Age Group**	**HI**
	**0–6 months babies**	
Brand 01	Female	0.0836
	Male	0.0774
Brand 02	Female	1.046
	Male	0.967
Brand 03	Female	0.0208
	Male	0.0192
Brand 04	Female	0.0094
	Male	0.0086
Brand 05	Female	0
	Male	0
Brand 06	Female	0
	Male	0
	**7–12 months babies**	
Brand 01	Female	0.686
	Male	0.0636
Brand 02	Female	0.858
	Male	0.79
Brand 03	Female	0.017
	Male	0.015
Brand 04	Female	0.007
	Male	0.007
Brand 05	Female	0
	Male	0
Brand 06	Female	0
	Male	0

Brands 01, 03, and 04 show safe levels of bisphenol consumption for 0–6-month-old newborns because their Hazard Quotient (HI) values are less than 1 for both male and female infants. However, Brand 02 may pose a risk because, for both genders in this age group, its HI values surpass 1, indicating a higher consumption of bisphenols than is deemed safe. While Brands 01, 03, and 04 maintain acceptable levels of bisphenol consumption with HI values below 1 for both genders, Brand 02 for female infants aged 7 to 12 months shows a potential concern with an HI value more than 1. Brand 05 and 06 continuously display HI values of 0, meaning that the consumption of these brands' milk will have no or negligible bisphenol leaching.

### 3.5 Estrogenic Activity Assessment

Estrogenic activity of milk kept in baby bottles for the analysis of BPA leaching was calculated and the results are presented in Table 8. Estrogenic potency (μg/kg/day) = 0.0000160 (Wang et al., 2020).

**Table 8:** Estrogenic activity assessment of Bisphenols in milk samples.

**Brand names**	**Estrogenic potency**	**EEQ μg/kg/day**
Brand 01	0.0000160 μg/kg/day	0.00000256
Brand 02		0.000032
Brand 03		0.00000064
Brand 04		0.000000288
Brand 05		0
Brand 06		0

Estrogenic potency results indicate which compounds in the milk samples may have effects similar to those of estrogen. Higher numbers imply a larger potential for estrogenic effects, whilst lower values indicate lesser estrogenic activity. With a 0.000032 μg/kg/day estrogenic potency value, Brand 02 has the highest potential for estrogen-like effects when compared to the other brands under analysis. The estrogenic potency values of Brands 01, 03, and 04 range from 0.0000160 to 0.00000064 μg/kg/day, showing different degrees of estrogenic activity. Of these brands, Brand 01 has the highest value. Estrogenic potency ratings for brands 05 and 06 are zero, indicating that there is no discernible estrogenic activity in these milk samples. It is important to considers these chemicals' possible estrogenic effects, particularly in vulnerable groups like newborns.

The observed variations in BPA concentrations among different brands are noteworthy, as they underscore the importance of employing accurate analytical methods in assessing chemical contaminants. These findings also prompt further investigation into the sources and potential health implications of elevated BPA levels in specific brands.

The detection of BPA leaching from plastics was first discovered in the early 1990s when it was found that yeast culture media exhibited estrogenic activity not originating from that culture media, but from polycarbonate flasks. The primary mode of exposure is dietary, which includes consuming food from contaminated regions, as well as foods packaged in plastics and cans (Basak, Das, & Duttaroy, 2020; K Cwiek-Ludwicka, 2015).The Bisphenol-A signal can be observed at around 5 min for the analysis of the standard solution and between 5 and 6 min for the migration sample during HPLC. In the analysis of specific migration, the Bisphenol-A peak obtained is distinct, symmetrical, and fully isolated from other analyte peaks. Quantitative analysis was conducted using an HPLC-UV detector and it is evident that the retention time for the samples with detected BPA levels showed peak eluting between 4.5 and 5.8 min. Chromatograms obtained for certain other analyzed brand samples did not exhibit any peaks near the retention time of BPA, as the response was below the limit of detection or ND for these samples, indicating they had no BPA content. Previous studies have indicated that the BPA peak occurs at a wavelength of 270 nm (Eckardt, Benisch, & Simat, 2020).

Chemical migration from food packaging into food is influenced by several factors. These include the properties and composition of the food, initial migrant concentration in the packaging, polymer matrix state, and migrant components in contact with the food. Additionally, storage duration, temperature, packaging size, contact period, and the ratio of packaging surface area to food volume play significant roles. Higher migrant concentrations in food over time suggest a faster transfer rate from packaging to food (Li et al., 2024; Sharafi et al., 2023). Moreover, our study observed significant variations in BPA levels when raw milk was exposed to different storage conditions. These findings are consistent with other studies indicating that BPA can migrate from both PC bottles and plastic storage containers that come into contact with milk and other dairy products(Vilarinho et al., 2019).

Polymer surface hydrolysis is the main process that releases BPA from polycarbonate (PC) into interacting aqueous liquids. The ozone concentration, temperature, and pH of the liquids, together with the PC surface's age, are important variables that affect this process (Mercea, 2009). Biologically active BPA is released from polycarbonate bottles into their contents during simulated regular usage, according to a number of prior research (Brede, Fjeldal, Skjevrak, & Herikstad, 2003; Le et al., 2008).

Furthermore, the repeated use and the heat exposure exhibited the escalation of leaching due to degradation of plastic polymer. (Farooq, Jalees, Hussain, Anis, & Islam, 2021; Onn Wong, Woon Leo, & Leng Seah, 2005; Santonicola et al., 2018).

Subsequent studies have consistently confirmed the detectable levels of BPA leach from plastic bottles. For instance, Farooq et al. (2021). noted the BPA leaching from polycarbonate bottles, whereas no such leaching was observed with glass bottles upon initial use. Recent LLE analysis reveals notably high mean concentrations of BPA in raw milk samples (brands 3 and 9) stored under specific temperature and duration conditions. In contrast, UHT and pasteurized milk samples exhibited lower BPA levels. These findings align with reported BPA leaching concentrations, influenced by various storage parameters, particularly in raw milk (PC packaging) (Farooq et al., 2021; Mercogliano, Santonicola, Albrizio, & Ferrante, 2021). As previously noted in earlier investigation, certain plastic containers may accelerate BPA leaching, depending on the degree of temperature utilized, while milk is kept in container (in- bottle sterilizing process) or in a bigger vat before bottling (UHT) (Biedermann-Brem & Grob, 2009; Casajuana & Lacorte, 2004).

The other findings from the present study using SPE that the concentration level of bisphenol A was higher in Tetra Pak milk containers compared to pasteurized milk. Additionally, pasteurization involves lower heat treatment than UHT, further reducing the risk of BPA leaching. Tetra Pak cartons typically include plastic linings that may contain BPA, which could gradually leach into the milk, especially under warmer conditions or with prolonged storage. Additionally, various studies have highlighted that variations in processing temperatures, composition of the packaging, processing methods, and the quality of carton sealing also influence the amount of BPA that migrates into the milk (Fayed, El Sheikha, Ali, & Hassan, 2023).

Moreover, the levels of contaminants in milk may vary based on geographic region and the class of chemicals analyzed, while their distribution in milk is influenced by the physicochemical properties of the chemical, applied processing methods, and the final milk product (Basak et al., 2020; K Cwiek-Ludwicka, 2015; Shelver, Lupton, Shappell, Smith, & Hakk, 2018). Mercogliano 2021 addressed about how different steps in the dairy company's milk processing may have led to varied amounts of contamination. These include the movement of processed milk from the homogenizer to the pasteurizer and filler packing equipment, as well as possible cross-contamination from lingering residues in raw milk storage (Mercogliano et al., 2021). BPA migration from plasticized contact materials, such as milk tubes and sealants used in the milking machine, as well as from the cooling tank's contact materials, may have contributed to the contamination levels detected in raw milk (Danaher & Jordan, 2013).

Generally, variations in BPA content is affected by its partitioning between milk fat and skim milk, which is governed by the compound's lipophilicity. Since BPA is fat-soluble, its concentration can vary depending on the milk fat content and quality of packaging material (Mercogliano & Santonicola, 2018; Shelver et al., 2018). However, the impact of these endocrine disrupting compounds on organisms and human beings needs to be further studied, especially with regard to accumulation, degradation, and possible effects within the endocrine system.

BPA has a range of harmful effects. It is categorized as a reproductive toxicant, with the potential to impair fertility or impact the developing fetus. Because of its potential to alter hormones, the European Chemical Agency listed BPA as a substance of very high concern (Andújar et al., 2019). The polymerization of 2,2-bis(4-hydroxyphenyl) propane, or bisphenol A (BPA), with phosgene yields polycarbonate (PC). The demonstration of BPA migration from PC products Concerns concerning the safety of using BPA in the manufacturing of PC food-contact goods have been raised by evidence of BPA migration from PC products as well as contentions of harmful effects on development and reproduction from low concentrations of BPA (Biles, McNeal, Begley, & Hollifield, 1997; Vom Saal et al., 1998).

Children, toddlers, and infants are particularly susceptible to EDCs. Exposure to BPA before and after childbirth has been associated with increased risks of cognitive problems, metabolic diseases such as obesity, and breast or prostate cancer in later life (Lucarini et al., 2020). Certain studies revealed that urinary BPA levels surged by two-thirds following just one week of using polycarbonate bottles (Carwile et al., 2009).

Toxicokinetics (TK) offers vital information about the BPA exposure evaluation with hazard toxicity analyses to identify possible dangers associated with human exposure to chemical substances. The toxicity order of these bisphenols is in the following order: BPAF > BPA = BPF > BPS > BPC > BPB > BPAP > BPZ (Catron et al., 2019). This study assessed potential toxicity and migration extent by correlating increased BPA levels in packaging with various factors. Table 3 presents the frequency of BPA detection as the percentage of positive samples by matrix. Investigation of raw milk samples, particularly those subjected to specific conditions, revealed a higher BPA BPA content in UHT as compared to pasteurized milk. However, earlier research has reported increase in BPA mean concentration, reaching 0.049 μg/ml and constituting (4.9 %) of its content, is observed in raw milk (Rykowska & Wasiak, 2006).

Our outcomes showed that PC containers were more contaminated with BPA than Tetra Brik and HDPE containers. These findings were further supported by other research that also demonstrated that PC baby bottles and PC water bottles had higher BPA contents (Brede et al., 2003; Cao et al., 2011; Liu, Ji, Zhang, & Liu, 2008).

Previous findings elaborated the consistent BPA levels (0.02 μg/L) in Norwegian cardboard-packaged milk (Russo, Barbato, Mita, & Grumetto, 2019) and higher levels in canned milk (Mercogliano et al., 2021). Earlier studies indicated the BPA contamination levels (0.035–2.776 μg/L) found in raw milk from the storage tank at the farm (Santonicola, Ferrante, Murru, Gallo, & Mercogliano, 2019).

Earlier analysis data also demonstrated that elevated temperatures and prolonged testing durations resulted in increased BPA migration from PC bottles (Kubwabo et al., 2009).

It was observed that as compared to pasteurized and cardboard-packaged milk, raw milk from the storage tank had the highest mean BPA contamination levels (0.265 μg/L), as reported by a subsequent literature study. Moreover the BPA concentrations in UHT milk packaging in HDPE from <10–62.0 μg/L (Grumetto et al., 2013; Mercogliano et al., 2021) and these results align with our findings. Pasteurized milk packed in PE containers was found to have BPA levels ranging from 23.41 to 41.69 μg/L (Abdulazeez, Yazici, Aksoy, & Tokur, 2022). Thus, our results agree with these reported values in milk stored in various containers but the concentration decrease in the order as raw milk (PC) > UHT milk(tetra-pak) > pasteurized milk (PE). The BPA residues observed above in recent analysis are highly comparable with previously reported values.

BPA is a Food Contact Material (FCM), which means that it is utilized in the manufacturing of polymers for items like plastic containers that have direct contact with food (Kazimiera Cwiek-Ludwicka & Ludwicki, 2014).

As a plasticizer and essential ingredient in epoxy resin and polycarbonate plastic manufacturing, bisphenol A (BPA) is widely used (Huang et al., 2012).

As, milk is a primary source of nourishment for individuals of all ages, making the detection of BPA in milk samples crucial. Even while the consequences of exposure to individual, endocrine disruptors have been the subject of several research, the potential effects of combinations of multiple toxic substances leached from plastic containers and their synergistic effects on human health are entitled to further consideration (Carnevali et al., 2017; Kolatorova, Duskova, Vitku, & Starka, 2017). The analysis of bisphenol A concentration in milk samples in this research work provides valuable information about migration extent and helps draw conclusions about the safety of storing milk in plastic containers. Furthermore, analyzing BPA levels encourages healthier as well as sustainable packaging practices and promoting innovation (Amir, Bano, Zaheer, Haq, & Roohi., 2022). These implications also highlight the potential significance of our work in advancing both health safety assessments and environmental considerations, particularly in estimating BPA levels in milk stored in various types of plastic containers. However extensive investigations are the need of hour to explore the presence of other possible bisphenol alternatives and promote the use of sustainable or BPA-free plastic materials for food storage.

Monitoring BPA levels is crucial to prevent potential health risks, particularly for infants. This study examines the issue of hazardous contamination related to BPA, focusing on the latest analytical methodologies for its detection and the BPA levels found in milk samples stored in different types of plastic containers, including PE, Tetra Pak, and polycarbonate. Research has suggested that BPA exposure can disrupt endocrine function and negatively impact human health, especially in infants.

Milk is a vital source of nutrition for babies, and it has been observed that polycarbonate (PC) containers, commonly used for baby bottles, are particularly prone to BPA contamination. Studies have highlighted that PC bottles used for infants often have higher BPA levels, which can pose risks to their endocrine system.

Though the multifaceted factors influencing BPA migration into the milk, such as composition of packaging materials, sterilization methods, temperature and storage duration, which are briefly discussed in this study. A careful selection of the various milk brands was made, based on the variety of their packaging materials to capture the variability present in real-world scenarios. This diversity was intended to provide a broad overview rather than focusing on a single variable. However, acknowledgment of the limited number of samples in the current study constrained to establish definitive correlations between BPA migration and the variables mentioned but this study was exploratory in nature and the present findings serves as a preliminary investigation that highlights the need for further research with extensive sample sizes, with emphasize on specific variables to draw more robust conclusions.

To address these concerns, a comprehensive study was conducted on various types of PC containers to explore the problems associated with their use thus highlighted the issues regarding BPA contamination in baby bottles and emphasize the importance of safe packaging for infant nutrition and development. Therefore, a comprehensive study should be organized to further investigate the polycarbonate plastic used in the manufacturing of storage containers, specifically in baby bottles that displayed maximum BPA leaching to address the harmful effects associated with early development mostly babies and also reproductive system.

## 4 Conclusion

Bisphenol A is a contaminant of significant concern due to its adverse effects on health and the environment, notably as an endocrine disruptor. BPA leaching from containers into milk samples was analyzed, employing a combination of SPE, LLE and HPLC-UV. Investigations showed the highest BPA leaching into raw milk samples, particularly those subjected to specific conditions. It is evident that UHT Milk packaging displayed the more BPA levels compared to pasteurized milk, as observed in both the LLE and SPE methods but the raw milk packaging demonstrated the highest BPA migration (at 70 degree Celsius) i.e., 0.049 μg/ml. These findings indicate that BPA leaching into milk from plastic packaging follows the trend as raw milk > UHT > pasteurized milk. BPA leaching into milk from plastic baby bottles at various temperature was among the major goals of current study.

Studies have highlighted that PC bottles used for infants often have higher BPA levels, which can pose risks to their health. To address these concerns, a comprehensive study was conducted on various types of PC containers to explore the problems associated with their use. High Bisphenol A (BPA) exposure may negatively impact on babies' health. Some bottles are BPA free while Brand No. 2 has a slightly high concentration of HI, which could lead to health problems in babies. To guarantee the safety and wellbeing of newborns, responsible authorities should take these variables into account when choosing milk for them. Brands with lower BPA levels, HQ values below 1, and minimum or no estrogenic potency should be prioritized. The analysis of bisphenol A concentration in the various brands of baby bottles and the milk samples stored in different types of containers provides valuable information about migration extent and aid in drawing conclusion about the safety of storing various food products and especially milk in plastic containers but the limited number of samples may not include the full range of milk products, available in the market which may affect the generalizability of the current findings. This limitation could affect the generalizability of our findings and since the samples were collected from a specific region, these findings may not be considered applicable to the other regions having different regulatory authorities and manufacturing standards. To improve the applicability of current findings extended sample size from diverse regions is recommended. Furthermore, analyzing BPA levels encourages healthier and sustainable packaging practices. In order to comprehend the long-term effects of exposure to such chemicals on newborn health, more investigation and observation are required.

## CRediT authorship contribution statement

**Ghulam Mustafa Kamal:** Writing – review & editing, Supervision, Resources, Project administration, Conceptualization. **Iqra Anwar:** Writing – original draft, Investigation, Formal analysis. **Kainat Saadullah:** Writing – original draft, Investigation, Formal analysis. **Attila Gere:** Visualization, Software, Funding acquisition. **Samra Yasmin:** Writing – original draft, Validation, Methodology. **Jalal Uddin:** Writing – review & editing, Visualization, Validation, Funding acquisition. **Abdullah Ijaz Hussain:** Methodology, Investigation. **Gulzar Ahmad Nayik:** Writing – review & editing, Visualization, Validation.

## Declaration of competing interest

The authors declare that there are no known competing financial interests or personal relationships that could have appeared to influence the work reported in this paper.

## Data Availability

Data will be made available on request.
